# Females Are More Sensitive to Opponent’s Emotional Feedback: Evidence From Event-Related Potentials

**DOI:** 10.3389/fnhum.2018.00275

**Published:** 2018-07-10

**Authors:** Xuhai Chen, Hang Yuan, Tingting Zheng, Yingchao Chang, Yangmei Luo

**Affiliations:** Key Laboratory of Behavior and Cognitive Psychology in Shaanxi Province, School of Psychology, Shaanxi Normal University, Xi’an, China

**Keywords:** gender difference, interpersonal emotion, decision making, RewP, feedback P300

## Abstract

It is widely believed that females outperformed males in emotional information processing. The present study tested whether the female superiority in emotional information processing exists in a naturalistic social-emotional context, if so, what the temporal dynamics underlies. The behavioral and electrophysiological responses were recorded while participants were performing an interpersonal gambling game with opponents’ facial emotions given as feedback. The results yielded that emotional cues modulated the influence of monetary feedback on outcome valuation. Critically, this modulation was more conspicuous in females: opponents’ angry expressions increased females’ risky tendency and decreased the amplitude of reward positivity (RewP) and feedback P300. These findings indicate that females are more sensitive to emotional expressions in real interpersonal interactions, which is manifested in both early motivational salience detection and late conscious cognitive appraisal stages of feedback processing.

## Introduction

Females are believed to have superiority in emotional competence such as understanding other people’s emotions embedded in facial cues (Hall, 1978; Hall and Matsumoto, 2004; Kret and De Gelder, 2012; Sawada et al., 2014; Weisenbach et al., 2014), even among adolescents and infants (McClure, 2000; Lee et al., 2013). However, it remains unclear whether this advantage extends to real interpersonal interactions, as the participants in previous studies were required to recognize emotions from static images without a naturalistic social-emotional context (Hall, 1978; Filkowski et al., 2017). This is of great importance given that decoding of emotional information always takes place in a specific context (Fukushima and Hiraki, 2006; Jack and Schyns, 2015; Wiggert et al., 2015; Pádua Júnior et al., 2016). Therefore, the present study approached this issue by recording behavioral and electrophysiological responses while participants performing an interpersonal gambling game with opponents’ facial emotions given as feedback (Chen et al., 2017).

It is widely reported that females are more sensitive to facial emotions in comparison with males (McClure, 2000; Donges et al., 2012; Erol et al., 2013; Lee et al., 2013; Weisenbach et al., 2014). For instance, females were more accurate in the categorization of fearful expressions relative to males in facial emotion perception test (Weisenbach et al., 2014) and females’ judgments of distance were more likely to be influenced by facial emotions (Kim and Son, 2015). And such behavioral advantage was also observed in adolescence, with girls more sensitive to facial emotions than boys (Lee et al., 2013). This female superiority in emotion decoding was also observed at subliminal level. For example, in subliminal affective priming experiment, Donges et al. (2012) reported that females manifested greater affective priming due to happy faces than males did. Likewise, Hoffmann et al. (2010) found that females were more accurate than males in recognizing subtle facial displays of emotion. Moreover, such female advantage in facial emotion recognition extends to other materials like voice (Demenescu et al., 2014; Lambrecht et al., 2014), point light displays (Alaerts et al., 2011), music (Hunter et al., 2011) and multisensory emotion expressions (Collignon et al., 2010).

Corresponding to the behavioral performance, the female superiority in emotion decoding is associated with different neural pathways and varied neurodynamics. A recent meta-analysis study showed that the medial prefrontal cortex, anterior cingulate cortex, frontal pole and the thalamus were more recruited in men relative to women during emotion perception, while women showed distinct activation in bilateral amygdala, hippocampus and some regions of the dorsal midbrain (Filkowski et al., 2017), suggesting that males tend to recruit bilateral prefrontal regions involved in rational thinking and cognitive control whereas females tend to recruit bilateral amygdala involved in quick emotional evaluation (AlRyalat, 2017). Regarding neurodynamics, it was reported that females yielded significantly larger P100 to fearful faces than males in emotion discrimination task (Lee et al., 2017), and generated longer latency and higher amplitude P450 component than males when explicitly detecting happy and sad faces among neutral faces (Orozco and Ehlers, 1998), suggesting that female advantage in emotion processing emerges in the early stage of low-level visual feature processing and the late stage of indepth emotionality evaluation. Likewise, females (but not males) yielded conspicuous N200 and P300 responses to moderately negative pictures (Li et al., 2008; Yuan et al., 2009) and demonstrated enhanced N200 when viewed unpleasant stimuli (Lithari et al., 2010), implying that gender difference in emotion decoding prevails in the initial perceptual coding and the deliberative categorization of the emotional expressions. Furthermore, Güntekin and Başar (2007) found that females generated significantly larger occipital beta responses (15–24 Hz) than males during the presentation of face expressions and argued that beta synchronization might mediate the female advantage in emotion processing.

The studies reviewed above revealed important insight into the female superiority in emotion processing. However, to the best of our knowledge, the influence of contextual factors on emotion processing has largely been neglected, despite these factors exert great impact on how observers ultimately discern facial expressions (Barrett et al., 2011; Kring and Campellone, 2012). Therefore, we hope to shed light on this issue by using the interpersonal version of Gehring and Willoughby’s gambling task (Gehring and Willoughby, 2002; Chen et al., 2017), in which participants chose between two monetary options and received feedback orthogonally combined monetary cues and emotional cues (Vrtička et al., 2014). Using this interpersonal paradigm, we hope to probe into the female advantage in emotion processing in naturalistic context concurrent with emotional and monetary feedback.

The neurophysiological studies of feedback processing focused on two event related potential (ERP) components. One is the frontocentral peaking component roughly 250–300 ms after feedback, which is thought to reflect early evaluation of performance feedback and action monitoring (Zhou et al., 2010; Ullsperger et al., 2014; Proudfit, 2015; Sambrook and Goslin, 2015). It was originally linked to negative feedback and referred as feedback related negativity (FRN; Gehring and Willoughby, 2002; Yeung et al., 2004), however, more recent research has indicated that the FRN effect may rather be driven by a reward positivity (RewP), which attenuates a default frontocentral N2 component and which is present for positive but not for negative outcomes (Proudfit, 2015; Heydari and Holroyd, 2016). The other is feedback-related P300, a positive deflection with parietal distribution occurring between 300 ms and 600 ms after feedback. This positive component, linking with a more elaborated and conscious appraisal of the motivational significance of performance feedback, was reported to be larger for positive feedback in comparison with negative feedback (Yeung et al., 2004; Leng and Zhou, 2010; Li et al., 2010; Ulrich and Hewig, 2014; Mason et al., 2016; Zhao et al., 2016). Moreover, these two components are sensitive to both monetary and emotional feedback, as the previous study reported that emotional and monetary reward elicited morphologically similar RewP (Ethridge et al., 2017) and feedback-related P300 (Oumeziane et al., 2017).

Given that emotion cues can bias decision-making (van Kleef et al., 2004; Averbeck and Duchaine, 2009; Parkinson et al., 2012; Chen et al., 2017), we predicted that emotional cues would interact with monetary cues in feedback processing. Specifically, opponents’ angry expressions should increase risky tendency and decrease RewP and feedback-related P300 associated with wins and losses, while happy expressions demonstrate the opposite effect if interpersonal emotion exerts its influence through affective reaction (van Kleef, 2009). Moreover, considering that females outperform males in emotion decoding (Hall, 1978; Hall and Matsumoto, 2004; Kret and De Gelder, 2012; Sawada et al., 2014; Weisenbach et al., 2014) and females are believed to be more interpersonally sensitive than men (Briton and Hall, 1995; Spence et al., 1975), we hypothesized the modulation of interpersonal emotions was more conspicuous in females relative to males.

## Materials and Methods

### Participants

Fifty right-handed university students (25 females) were recruited to participate in this experiment. All participants reported normal auditory and normal or corrected-to-normal visual acuity and were free of neurological or psychiatric problems. Four participants (two females) were excluded from analysis due to excessive EEG artifacts in the recordings. The remaining participants showed no significant difference between genders on age, personality and emotional intelligence (EI; see Table 1 for illustration). This study was carried out in accordance with the recommendations of the Declaration of Helsinki. The protocol was approved by the the Ethical Committee of Shaanxi Normal University. All subjects gave written informed consent in accordance with the Declaration of Helsinki.

**Table 1:** Age, personality and emotional intelligence* of the participants as a function of gender.

	Male (*n* = 18)	Female (*n* = 18)	*t*	*P*
Age	19.52 ± 1.13	19.08 ± 1.02	−1.42	0.16
Neuroticism	2.95 ± 0.49	2.68 ± 0.48	1.87	0.07
Extraversion	3.27 ± 0.51	3.32 ± 0.40	−0.34	0.73
Openness	3.18 ± 0.58	3.26 ± 0.68	−0.43	0.67
Agreeableness	3.19 ± 1.21	2.93 ± 0.61	0.52	0.61
Conscientiousness	3.34 ± 0.46	3.37 ± 0.41	−0.25	0.80
EI	3.71 ± 0.26	3.83 ± 0.34	−1.39	0.17

*Personality were assessed with shortened Chinese version of the Costa and McCrae NEO-FFI (Yang et al., 1999), while emotional intelligence (EI) were assessed with ESI (Schutte et al., 1998).

### Procedure

Upon entry into the lab, the participant was introduced to a confederate of the same gender who would perform as opponent in a gambling game through a computer network. They were told they would played as competitors, that is to say, a loss for the participant means a win for his/her opponent in the same amount, and vice versa. And then their facial expressions (happy, angry and neutral) were recorded using a Canon EOS 600D and used as feedback stimuli. Unbeknownst to the participant, the facial expression of the confederate was prerecorded and validated in advance. Immediately after giving informed consent, participants were endowed with ¥40. They were told the money was theirs to risk during the study and asked to place it in their wallets. Participants were told that additional rewards or punishments were given based on their performances. The actual earnings for the each participant ranged from ¥30 to ¥50.

**Figure 1:**
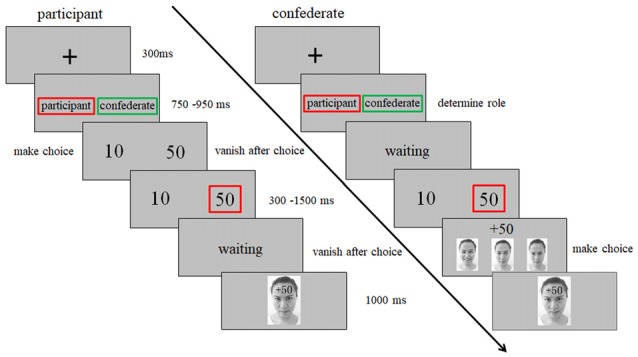
Schematic diagram of an experimental trial in the interpersonal gambling task. After a fixation, the computer selected performer (red square) and observer (green square) for each round of gambling randomly. The person selected as the performer would view numeral 10 or 50 (cents) and make a choice by pressing the corresponding button as soon as possible. After the choice presented for 300–1500 ms randomly, the observer saw the monetary outcome and chose one of his/her facial expressions to indicate his/her attitude: happiness means he/she is happy with the outcome, anger means he/she is angry with the outcome, neutral means no specific emotion. Then, the selected facial expression overlaid with the monetary cues (“+50” or “−50”) on the forehead were presented as feedback for 1000 ms. While “+” means won, “−” means lose.

Following our previous study (Chen et al., 2017), the task in the present study was adapted from Gehring and Willoughby gambling task (Gehring and Willoughby, 2002). The key adaption was the interactivity (Chen et al., 2017) and the feedback which orthogonally combined with monetary and emotional cues (Vrtička et al., 2008, 2014; Chen et al., 2017). Figure 1 shows a schematic diagram of a trial in this task. Specifically, after a fixation period, the participants were told that the computer would select performer and observer for each round of gambling randomly. The person selected as the performer would view numeral 10 or 50 (cents) and make a choice by pressing the corresponding button as soon as possible. After the choice presented for 300–1500 ms randomly, the observer saw the monetary outcome and chose one of his/her facial expressions to indicate his/her attitude: while happiness means he/she is happy with the outcome, anger means he/she is angry with the outcome, and neutral expressions means no specific emotions. Then, the selected facial expression overlaid with the monetary cues (“+50” or “−50”) on the forehead were presented as feedback for 1000 ms. While “+” indicated that the performer won the points, “−” indicated the performer lose the points. Unbeknownst to the participant, the monetary outcomes and affective responses of the confederate were predetermined. Each participant was selected as performer two thirds of the trials and as observer the remaining third. Each participant received four types of feedback (happy-win, happy-lose, angry-win and angry-lose) equally with 64 trials. To make the game more realistic, 32 neutral-win and 32 neutral-lose trials were included as fillers, which were not included in the data analysis. The whole experiment consisted of 448 trials, dividing into eight blocks with 56 trials each.

### EEG Recording

EEG measurements were recorded at 64 scalp sites using tin electrodes mounted in an elastic cap (Brain Product, Munich, Germany) according to the modified expanded 10–20 system, each referenced online to FCZ. Vertical electrooculogram (EOG) was recorded supra-orbitally and infra-orbitally from the right eye. The horizontal EOG was recorded as the left vs. right orbital rim. The EEG and EOG measurements were amplified using a 0.05–100 Hz bandpass and continuously digitized at 1000 Hz for offline analysis. The impedance of all electrodes was kept less than 5 kΩ.

### Data Analysis

#### Preprocessing

**Figure 2:**
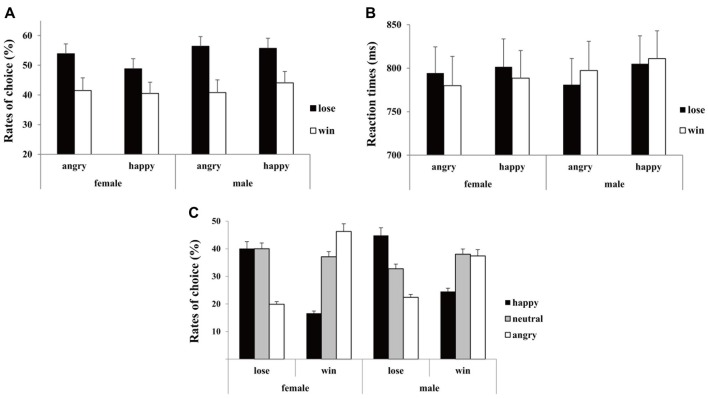
Behavioral performance. **(A)** Mean rates of risky choice, **(B)** the corresponding reaction times (RTs) and **(C)** rates of emotional feedback selection as a function of conditions for females and males separately. Error bars indicate standard error.

The “10” is defined as the low-risk option (small potential win or loss) while the “50” is defined as the high-risk option (large potential win or loss). The risk-seeking preference was measured as the “risk ratio” by dividing the number of high-risk choices by the total number of choices. Following previous studies (Gehring and Willoughby, 2002; Chen et al., 2017), we analyzed the preceding outcome on risky behavior in the current trial. Thus, the risk ratio of the second trial during consecutive trials and the corresponding reaction times (RTs; beyond three standard deviations were excluded in RT calculation) were calculated as the dependent variable (see Figure 2).

**Figure 3:**
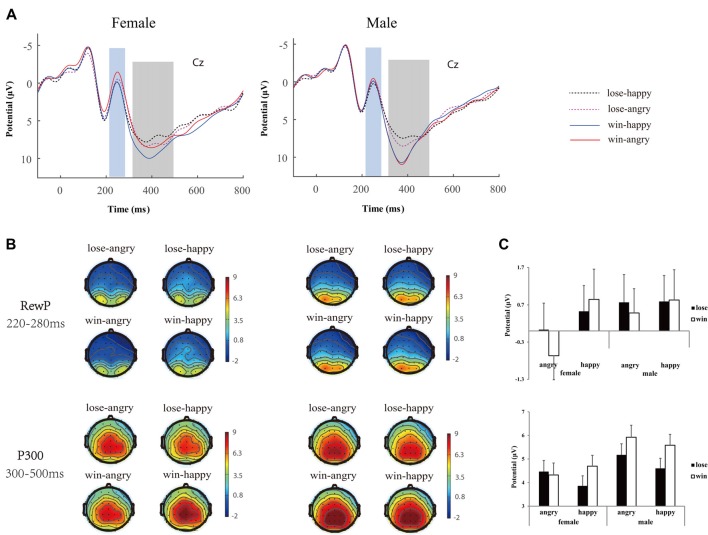
Neurophysiological results. **(A)** Group-averaged event related potential (ERP) voltage waveforms over Cz, **(B)** scalp topography (top view shown) and **(C)** bar plots of mean ERP values for reward positivity (RewP) and P300 during the selected time window as function of conditions. Error bars indicate standard error.

EEG data was preprocessed using EEGLAB (eeglab13_6_5b), an open source toolbox running on the MATLAB platform (R2014a). First, the data were high pass filtered at 0.5 Hz, and re-referenced offline to bilateral mastoid electrodes. The data were segmented into epochs around the presentation of outcome feedback stimuli (−200 to 800 ms post stimulus). The epoched data were baseline corrected using 200 ms before the onset of the feedback. EEG epochs with large artifacts (exceeding ±100 μV) were removed, and channels with poor signal quality were interpolated spherically using EEGLAB toolbox (Perrin et al., 1989). Trials contaminated by eye blinks and other artifacts were corrected using an independent component analysis algorithm (Delorme and Makeig, 2004). There were on average 59.89 ± 4.17, 59.46 ± 3.71, 59.75 ± 4.48 and 59.21 ± 4.90 artifact-free trials obtained for the lose-angry, lose-happy, win-angry and win-happy conditions for females, while 58.83 ± 2.91, 58.61 ± 2.47, 57.56 ± 3.18 and 58.44 ± 3.27 remained for males. Note that the magnitude (10 vs. 50) of the outcome was collapsed for conciseness. After low-pass filtered at 30 Hz, extracted average waveforms for each participant and condition were used to calculate grand-average waveforms. For statistical analyses, following previous studies (Calvo and Beltrán, 2013; Chen et al., 2017), the mean amplitude between 220 ms and 280 ms over fronto-central cluster (F1, Fz, F2, FC1, FCz, FC2, C1, Cz, C2) was calculated for RewP, whereas the mean activity between 300 ms and 500 ms at the parietal cluster (P1, Pz, P2, PO3, POz, PO4) was calculated to assess feedback P300 (see Figure 3).

### Statistical Analysis

We entered the behavioral data and ERP data into repeated measures ANOVAs, with the *outcome valence* (loss vs. win) and *emotion* (happy vs. angry) as within-subject factors and *gender* (male vs. female) as a between-subject factor. To examine how participants take the current interpersonal gambling task, the rates of each emotional expressions they chosen as feedback were analyzed accordingly. The degrees of freedom of the F-ratio were corrected per the Greenhouse–Geisser method, and multiple comparisons were Bonferroni adjusted if necessary. The effect sizes are shown as partial eta squared ($\eta_{\text{p}}^{2}$).

## Results

### Behavioral Performance

The participants selected more high-risk options after losses (0.54 ± 0.02) than after wins (0.42 ± 0.03), (*F*_(1,44)_ = 15.45, *p* < 0.001, $\eta_{\text{p}}^{2}$ = 0.26). Moreover, there were marginal significant two-way interaction between *emotion* and *outcome* (*F*_(1,44)_ = 3.29, *p* = 0.08, $\eta_{\text{p}}^{2}$ = 0.06), and marginal significant two-way interaction between emotion and gender (*F*_(1,44)_ = 3.88, *p* = 0.05, $\eta_{\text{p}}^{2}$ = 0.08). Simple effect analysis indicated that the difference in risky selection was more conspicuous following opponents’ angry expressions (win: 0.43 ± 0.04 vs. lose: 0.56 ± 0.03, *p* < 0.001) in comparison with happy expressions (win: 0.41 ± 0.04 vs. lose: 0.51 ± 0.03, *p* = 0.02; see Figure 2A). Additionally, while females’ choices tended to be affected by opponents’ expressions (happy: 0.45 ± 0.03 vs. angry: 0.48 ± 0.03, *p* = 0.05), males’ choices were immune to opponents’ expressions (happy: 0.50 ± 0.03 vs. angry: 0.49 ± 0.03, *p* = 0.39). The analysis on RTs only showed a significant main effect of *emotion* (*F*_(1,44)_ = 5.29, *p* = 0.03, $\eta_{\text{p}}^{2}$ = 0.11), with the RTs longer following opponents happy expressions (805 ± 22 ms) relative to angry expressions (788 ± 22 ms; see Figure 2B).

The analysis on rates of emotional expression only showed a two-way interaction between *emotion* and *outcome* (*F*_(2,88)_ = 10.11, *p* = 0.002, $\eta_{\text{p}}^{2}$ = 0.19). Simple effect analysis indicated that the angry expressions (0.21 ± 0.03) were less selected than both happy (0.43 ± 0.03, *p* = 0.01) and neutral (0.36 ± 0.04, *p* = 0.03) expressions if opponent lost the game, in contrast, happy expressions (0.21 ± 0.03) were less selected than both angry (0.42 ± 0.04, *p* = 0.01) and neutral (0.38 ± 0.04, *p* = 0.01) expressions if opponent won the game (see Figure 2C).

### Neurophysiological Performance

The analysis of RewP showed a main effect of *emotion* (*F*_(1,44)_ = 9.32, *p* = 0.004, $\eta_{\text{p}}^{2}$ = 0.18), a significant interaction of *emotion* × *gender* (*F*_(1,44)_ = 4.42, *p* = 0.04, $\eta_{\text{p}}^{2}$ = 0.09), and a marginal significant interaction of *emotion* × *outcome valence* (*F*_(1,44)_ = 3.32, *p* = 0.07, $\eta_{\text{p}}^{2}$ = 0.07). Simple effect analysis (see Figure 3) indicated females differentiated the angry (−0.32 ± 0.68 μV) from happy (0.69 ± 0.73 μV, *p* < 0.01) expressions, while males failed to do this (angry: 0.63 ± 0.68 μV vs. happy: 0.81 ± 0.73 μV, *p* = 0.53). Moreover, the RewP was more positive going following happy expressions (0.84 ± 0.57 μV) relative to angry expressions (−0.09 ± 0.46 μV, *p* = 0.002) if participants won the game, whereas the RewP was hardly differentiated between emotions (happy: 0.66 ± 0.49 μV vs. angry: 0.40 ± 0.52 μV, *p* = 0.31) if participants lost the game.

The analysis of P300 amplitudes showed a main effect of *outcome valence* (*F*_(1,44)_ = 25.22, *p* < 0.001, $\eta_{\text{p}}^{2}$ = 0.37), and a main effect of *emotion* (*F*_(1,44)_ = 7.77, *p* = 0.008, $\eta_{\text{p}}^{2}$ = 0.15). Also significant were the interaction of *emotion* × *outcome valence* (*F*_(1,44)_ = 12.98, *p* = 0.001, $\eta_{\text{p}}^{2}$ = 0.23), and a significant interaction of *outcome valence* × *gender* (*F*_(1,44)_ = 4.63, *p* = 0.03, $\eta_{\text{p}}^{2}$ = 0.10). Critically, the three-way interaction of *emotion* × *outcome valence* × *gender* is significant (*F*_(1,44)_ = 4.94, *p* = 0.03, $\eta_{\text{p}}^{2}$ = 0.10). Simple effects analysis yielded that, for the females, the wins (4.70 ± 0.46 μV) elicited larger P300 amplitudes than the losses (3.86 ± 0.43 μV, *p* = 0.001) when accompanied with happy expressions, while the P300 differences between wins (4.32 ± 0.51 μV) and losses (4.43 ± 0.47 μV, *p* = 0.44) were diminished when accompanied by angry expressions. In contrast, for the males, the wins elicited larger P300 than losses when accompanied by both angry (5.92 ± 0.51 μV vs. 5.17 ± 0.47 μV, *p* < 0.001) and happy (5.58 ± 0.46 μV vs. 4.60 ± 0.42 μV, *p* < 0.001) expressions.

## Discussion

To examine the female superiority of emotion decoding in a real social context, this study required participants to play an interpersonal gambling game with monetary and emotional cues orthogonally combined as feedback. The results yielded that participants selected more happy expressions for opponents’ losses but more angry expressions for opponents’ wins. Moreover, participants selected more high-risk options following losses relative to wins, and such effect was more conspicuous when accompanied with opponents’ angry expressions. Additionally, while females’ risky tendency was affected by opponents’ emotional feedback, males showed no such tendency. Corresponding to these behavioral results, RewP and feedback P300 for females was influenced by opponents’ emotional feedback, but not for males. The significance of these findings will be addressed as the following.

According to Emotions as Social Information Model (van Kleef, 2009), emotional expressions affect observers’ behavior by triggering inferential processes and/or affective reactions in them, consequently, emotional expressions can be used as strategy to influence observers’ behavior (Xiao and Houser, 2005). The participants in current study chose more happiness for opponents’ losses but more anger for opponents’ wins, indicating that they were aware of the setup of the experiment and used emotional expressions as tactics to affect opponents. In turn, we assumed that participants would take opponents’ emotional feedback seriously given that they performed strategically when they had the right to give emotional feedback. And thus, this result can evidence the good validity of our interpersonal gambling game.

In line with previous studies (Gehring and Willoughby, 2002; Yeung et al., 2004; Chen et al., 2017), participants were more likely to gamble on risky outcomes if on the previous trial they had lost the points. This might due to that participants were more willing to anticipate larger monetary rewards in order to reduce negative consequences. By contrast, they were more prone to protect the money they had and thus showed more conservative behavior when faced with rewarding feedback. Complement to the previous studies, the current study showed that the willingness to engage in risky choice following losses was affected by opponents’ emotional feedback. Specifically, opponents’ angry expressions enlarged the risky tendency relative to happy expressions. Given that angry and happy expressions, used as social feedback, could bring in similar effect as monetary feedback (Vrtička et al., 2014; Ethridge et al., 2017; Oumeziane et al., 2017), we speculate that the current modification result from the interaction of two types feedback cues. Moreover, combined with the influence of emotional cues on RTs, the current finding supported the assumption that interpersonal emotions bias ones’ decision making (van Kleef et al., 2004; Averbeck and Duchaine, 2009; Parkinson et al., 2012; Chen et al., 2017).

Consistent with these behavioral findings, we observed conspicuous interaction between monetary and emotional cues on both the RewP and feedback P300. This finding replicated the previous finding that opponent’s angry expressions reversed the differentiation pattern of RewP/FRN and diminished feedback P300 difference associated with losses and wins (Chen et al., 2017). Following previous studies (Chen et al., 2017; Proudfit, 2015; Heydari and Holroyd, 2016), we speculated that this phenomenon might result from that opponents’ angry expressions were taken as negative feedback and thus reduced the positive-going deflection elicited by wins. Moreover, the current finding was in accordance with the previous studies showing that interpersonal emotional expressions affect negotiation (van Kleef et al., 2004), dispute resolution (Friedman et al., 2004), cooperation (Krumhuber et al., 2007), and prosocial behaviors (van Doorn et al., 2015). Taken together, these findings provided evidence for the assumption that emotional information biases decision-making (Averbeck and Duchaine, 2009; Evans et al., 2011; Parkinson et al., 2012; Aïte et al., 2013). Complement to the previous studies, the current findings depicted the neurodynamics of the impact of interpersonal emotions. Given that RewP/FRN is associated with early evaluation of performance feedback and action monitoring (Gehring and Willoughby, 2002; Holroyd et al., 2008; Ullsperger et al., 2014; Proudfit, 2015) while P300 reflected elaborated appraisal of the motivational significance of outcome (Yeung et al., 2004; Leng and Zhou, 2010; Li et al., 2010; Ulrich and Hewig, 2014; Mason et al., 2016; Zhao et al., 2016), the current findings suggested that interpersonal emotions might affect outcome processing during both early stage of motivational salience monitoring and late stage of cognitive appraisal processing.

More critical to the current study, we found that females were more prone to be influenced by opponents’ emotional feedback. That is, opponents’ angry expressions increased females’ risk tendency, decreased RewP and feedback P300 in comparison with happy expressions. Given that angry expressions have been used as a negative social feedback (Vrtička et al., 2014; Ethridge et al., 2017; Oumeziane et al., 2017) and elicited smaller RewP (Ethridge et al., 2017) and feedback P300 (Oumeziane et al., 2017), the current findings suggested that females are highly susceptible to emotional feedback, and consquently modified the amplitude of RewP and P300. Based on the modulation on RewP and feedback P300, it seems the impact of angry expressions can even overshadow the influence of monetary cues during both early stage of motivational salience monitoring and late stage of cognitive appraisal processing for females. In contrast, for males, emotional feedback only counteracted effect of monetary cues during the early stage of salience monitoring, but not the late stage of in-depth valuation. This finding was in accordance with the neuroanatomical findings that while males tend to be rational by recruiting bilateral prefrontal regions, females tend to be emotional by recruiting bilateral amygdala when facing with emotional information (AlRyalat, 2017; Filkowski et al., 2017). Actually, females have long been believed to outperform males at recognizing emotions expressions (McClure, 2000; Li et al., 2008; Yuan et al., 2009; Donges et al., 2012; Erol et al., 2013; Lee et al., 2013; Weisenbach et al., 2014; Mason et al., 2016), and more prone to be influenced by emotional information (Schirmer et al., 2002, 2004; Kim and Son, 2015). The current conspicuous female advantage of emotion decoding during both early stage of motivational salience monitoring (RewP) and late stage of cognitive appraisal processing (feedback P300) was in line with the findings that gender difference in emotions processing emerges at early stage of emotion extraction (Lee et al., 2017) and late stage of emotion in-depth processing (Orozco and Ehlers, 1998). Taken together, the current study provided convergent evidence for the gender difference in interpersonal emotion decoding, adding new knowledge to this area by taking the contextual factors into consideration (Barrett et al., 2011; Kring and Campellone, 2012).

Although the explanation of the influence of angry expression on feedback P300 is quite reasonable, the reverse of RewP/FRN for losses and wins is still elusive. However, this phenomenon seems to be robust, as we observed this pattern again (Chen et al., 2017). One quite possible reason is the congruency between emotional and monetary cues: the incongruence might result in more negative valence. To support this speculation, a study employed similar design reported that right inferior frontal gyrus was more activated for incongruent feedback than for congruent feedback (Vrtička et al., 2014). Another possible reason is that emotional feedback is so salient that overshadow the influence of monetary feedback. The big facial expressions overlaid with small monetary cues in the current study might also boost this tendency. However, all these speculations still need further studies.

Despite the contributions of this study, some limitations should be noted. First, the use of emotional expression taken from participants and confederates surely increase the ecological validity, however, the external validity might be constrained. Second, although we found conspicuous gender difference in interpersonal emotion decoding after controlling age, personality and EI, we did not take sex hormonal levels and menstrual cycle into consideration. Given that sex hormones and cycle phases are implicated in sexual dimorphism in facial emotion recognition (Derntl et al., 2008; Guapo et al., 2009), future studies should take these factors into consideration. Third, whether biological sex or psychological gender identity matter in gender difference (Bourne and Maxwell, 2010) in interpersonal emotion processing is also an interesting topic in future studies.

## Conclusion

The present study examined the gender difference in interpersonal emotions processing. Participants were asked to perform interpersonal gambling task with opponents’ emotional expressions presented as feedbacks. It was found that opponents’ angry expressions increased females’ risky tendency and decreased the amplitude of RewP and feedback P300. These findings indicate that females are more sensitive to emotional expressions in interpersonal interactions, which is manifested during early stage of motivational salience monitoring and late stage of conscious appraisal of outcomes.

## Author Contributions

XC, HY and TZ designed the study and discussed the results. HY, TZ and YC organized the studies, analyzed the data and wrote a first draft of the article, which was revised by YL.

## Conflict of Interest Statement

The authors declare that the research was conducted in the absence of any commercial or financial relationships that could be construed as a potential conflict of interest.
